# Gut microbiome structure and adrenocortical activity in dogs with aggressive and phobic behavioral disorders

**DOI:** 10.1016/j.heliyon.2020.e03311

**Published:** 2020-01-29

**Authors:** E. Mondo, M. Barone, M. Soverini, F. D'Amico, M. Cocchi, C. Petrulli, M. Mattioli, G. Marliani, M. Candela, P.A. Accorsi

**Affiliations:** a Department of Medical Veterinary Science, University of Bologna, Ozzano Emilia, Italy; b Unit of Holobiont Microbiome and Microbiome Engineering (HolobioME), Department of Pharmacy and Biotechnology, University of Bologna, Italy

**Keywords:** Biological sciences, Microbiology, Endocrinology, Veterinary medicine, Animal behavior, Microbiome, Behavioral disorders, Aggressive dogs, Phobic dogs, Hormones

## Abstract

Accompanying human beings since the Paleolithic period, dogs has been recently regarded as a reliable model for the study of the gut microbiome connections with health and disease. In order to provide some glimpses on the connections between the gut microbiome layout and host behavior, we profiled the phylogenetic composition and structure of the canine gut microbiome of dogs with aggressive (n = 11), phobic (n = 13) and normal behavior (n = 18). Hormones’ determination was made through Radio Immuno-Assay (RIA), and next generation sequencing of the V3–V4 gene region of the bacterial 16S rRNA was employed to determine gut microbiome composition. Our results did not evidence any significant differences of hormonal levels between the three groups. According to our findings, aggressive behavioral disorder was found to be characterized by a peculiar gut microbiome structure, with high biodiversity and enrichment in generally subdominant bacterial genera (i.e. *Catenibacterium* and *Megamonas*). On the other hand, phobic dogs were enriched in *Lactobacillus*, a bacterial genus with known probiotic and psychobiotic properties. Although further studies are needed to validate our findings, our work supports the intriguing opportunity that different behavioral phenotypes in dogs may be associated with peculiar gut microbiome layouts, suggesting possible connections between the gut microbiome and the central nervous system and indicating the possible adoption of probiotic interventions aimed at restoring a balanced host-symbiont interplay for mitigating behavioral disorders.

## 1 Introduction

Dogs were domesticated during the Paleolithic period, accompanying humans across the transition from hunting-gathering to rural agriculture of the Neolithic, to post-industrialized Western lifestyle (Larson et al., 2012; Wang et al., 2013; Frantz et al., 2016). The frequent sharing of food resources with human beings has been a selective force able to drive changes in the digestive and metabolic system of dogs, enabling them to efficiently adapt to a more starch-enriched diet compared to their wild ancestor, and ultimately influence canine behavior (Axelsson et al., 2013; Ollivier et al., 2016). The canine gastrointestinal tract hosts a complex and highly biodiverse microbial ecosystem, whose predominant taxa resemble those typically found in the gut of other omnivorous mammals. However, in comparison to both mice and pigs, the canine gut microbiome (GM) result the most similar to humans (Ley et al., 2008; Coelho et al., 2018). As observed in humans, the eubiotic and stable configuration of the canine GM is therefore of fundamental importance for the maintenance of a homeostatic gut environment and of the overall host health. Several recent studies have shown the ability of the mammalian GM to communicate with the host central nervous system (CNS) through several parallel channels, involving the vagus nerve, neuroimmune and neuroendocrine signaling mechanisms, and the production of neuroactive chemicals – i.e. gamma-aminobutyric acid (GABA), serotonin (5-HT), norepinephrine and dopamine (Lyte, 2013; Forsythe et al., 2014; Neuman et al., 2015; Fung et al., 2017). Conversely, the CNS can influence GM structure and metabolome, influencing the gut environment, acting on motility, secretion and permeability via the autonomic nervous system (ANS) (Mayer, 2011). It is thus a matter of fact that the GM can influence the host behavior and *vice versa*, exerting a key role in the modulation of the gut-brain axis. The development of researches during the last decades indeed suggests the presence of a bidirectional communication between gut and brain.

Dog's behavior disorders are common problem, which often can be causes of animals' abandonment. Animal shelters have to face with those problems, but sometimes the interventions are few and not always effective (Salman et al., 2000). Despite the high variability and severity of behavioral disorders observed in dogs, the aggressive behavior has been found to be the most common, followed by separation anxiety and phobia (Yalcin and Batmaz*.*, 2007). Aggressiveness is a personality trait of dogs influenced by both genetic and environmental components (such as socialization and learning), characterized by behaviors of threats (i.e. growling, raised tale, and bared teeth) and aggression (bite and attack) (Walker et al., 1997). Phobia is characterized by a long lasting and intense state of fear, which can hesitate in panic (Walker et al., 1997; Ibáñez and Anzola, 2009). Phobia and aggressiveness are both expression of anxiety disorders (Ibáñez and Anzola, 2009) and they are strictly correlate to distress conditions of animals (Apple and Pluijmakers, 2004; Rosado et al., 2010). Stress is a threat for organism homeostasis and the body responds with a series of physiological response, the activation HPA (hypothalamic-pituitary-adrenocortical) axis and the production of glucocorticoids (Beerda et al., 1997). Higher long-term stress level is a key component of the gut – brain axis and potentially it is affected by GM changes (Bravo et al., 2011). Although recent works on canine microbiome have investigated potential interactions with aggression, these studies have focused on the variations of their GM profile after targeted dietary interventions to reduce aggressive behaviors (Re et al., 2008; Kirchoff et al., 2018). To the best of our knowledge, no study has focused on the comparison of the GM structure between dogs exhibiting aggressive, phobic and normal behavior, with specific associations with adrenocortical activity. In order to provide some glimpses in this direction, our work aims to compare both the gut microbiota composition and the endocrine framework of dogs affected by behavioral disorders (aggressive and phobic animals) and healthy dogs. The importance of this study lies in the fact that new findings in this field can contribute to a better knowledge of the mechanisms that correlate gut microbiota and behavior, and to assess in what way problem behavior in dogs may be reduced through dietary means or new therapeutic procedures.

## 2 Materials and methods

### 2.1 Enrolled animals, behavioral evaluation and sample collection

The entire study was previously evaluated and approved by the Scientific Ethic Committee for Animal Experimentation (University of Bologna). All the procedures were monitored by the responsible of the Department of Veterinary Medical Science (DIMEVET) for animal welfare.

In the study, 42 dogs (23 males and 19 females) of different breed, age, and weight, housed in cages, alone or with other dogs, according to the rules of AUSL of Bologna, of three different animal shelters located in the metropolitan area of Bologna (Italy) were enrolled (Table 1). The animals were fed on a mixed diet, including wet and dry commercial feed and additional homemade food (pork, chicken, lamb, vegetable and fruit).

**Table 1:** Metadata of the enrolled cohort. Characteristics of groups composition: number, sex and mean age ±standard deviation (s.d.) of dogs enrolled in the study.

	*Normal behavior (N) n=18*	*Aggressive behavior (A) n=11*	*Phobic behavior (P) n=13*
*Male not sterilized*	4	4	4
*Male neutered*	4	5	2
*Female not sterilized*	4	0	2
*Female neutered*	6	2	5
*Mean age ± S.D. (years)*	6 ± 3.9	7 ± 3.4	6 ± 2.8

For each animal, a behavioral evaluation was conducted by a Behaviorist Veterinarian, which has classified the dogs enrolled according to their behavioral phenotype. The evaluation was conducted using the grid of Giussani et al. (2013). 11 animals were classified as aggressive and 13 as phobic, while 18 animals were considered healthy animals, which did not present any behavioral problems.

Fecal samples were collected from each animal immediately after the evacuation using sterile materials, avoiding debris and cross-contaminations, between 7:00 and 12:00 am. Specimens were frozen with liquid nitrogen and transported to the laboratory, then stored at -80 °C until DNA extraction and sequencing. The remaining part of fecal samples was picked up through non-sterile bags and frozen at -20 °C until cortisol and testosterone assay.

### 2.2 Fecal cortisol and testosterone Radio Immuno-assays

Cortisol and testosterone concentrations were determined by Radio Immuno-assays (RIAs) based on binding of ^3^H-steroid by competitive adsorption (Fenske and Schonheiter, 1991). All concentrations were expressed in pg/mg of fecal matter. Extraction methodology was modified from Schatz and Palme (2001). Cortisol and testosterone were extracted from fecal specimens (500 mg, wet weight) with methanol-water solution (v/v 4:1) and ethyl ether. The portion of ether was vaporized under an airstream suction hood at 37 °C. Dry residue was finally dissolved again into 0.5 ml PBS (0.05 M, pH 7.5). The extraction was performed as described before, yielding a mean percentage recovery of 87.5 ± 2.4 and 89.3 ± 2.1 for cortisol and testosterone, respectively. Cortisol and testosterone metabolites assay in feces were carried out according to Tamanini et al. (1983) and Gaiani et al. (1984), respectively. Analysis were performed in duplicates. The cortisol RIA was performed using an antiserum to cortisol-21-hemisuccinate-BSA (anti-rabbit), at a working dilution of 1:20 000 and ^3^H-cortisol (30 pg/tube vial) as tracer. The testosterone RIA was performed using an antiserum to testosterone-3-carboxymethyloxime-BSA (anti-rabbit), at a working dilution of 1:35 000 and ^3^H-testosterone (31 pg/tube vial) as tracer. Validation parameters of analysis were: sensitivity 0.19 pg/mg, intra-assay variability 5.9%, inter-assay variability 8.7%, for cortisol; sensitivity 1.1 pg/mg, intra-assay variability 6.2%, inter-assay variability 9.6%, for testosterone. Radioactivity was determined using a liquid scintillation β counter and a linear standard curve, ad hoc designed by a software program (Motta and Degli Esposti, 1981).

### 2.3 Bacterial DNA extraction from stool samples

Total microbial DNA was extracted from each fecal sample using the DNeasy Blood & Tissue kit (QIAGEN), with the modified protocol described by Barone et al. (2019). Briefly, 250 mg of feces were resuspended in 1 ml of lysis buffer (500 mM NaCl, 50 mM Tris-HCl pH 8, 50 mM EDTA, 4% SDS). Fecal samples were added with four 3-mm glass beads and 0.5 g of 0.1-mm zirconia beads (BioSpec Products, Bartlesville, USA) and homogenized with 3 bead-beating steps using the FastPrep instrument (MP Biomedicals, Irvine, CA) at 5.5 movements/s for 1 min, keeping the samples on ice for 5 min after each treatment. Samples were subsequently heated at 95 °C for 15 min and centrifuged to pellet stool particles. Supernatants were added with 260 μl of 10 M ammonium acetate, centrifuged for 10 min at full speed, and incubated in ice for 30 min with one volume of isopropanol. Nucleic acids were collected by centrifugation, washed with 70% ethanol and resuspended in 100 μl of TE buffer. RNA and protein removal were performed by incubating the samples with DNase-free RNase (10 mg/ml) at 37 °C for 15 min and proteinase K at 70 °C for 10 min, respectively. Subsequently, DNA purification with QIAmp Mini Spin columns were performed as per manufacturers instruction. The extracted bacterial DNA was quantified using the NanoDrop ND-1000 spectrophotometer (NanoDrop Technologies).

### 2.4 PCR amplification and sequencing

The V3–V4 region of the 16S rRNA was amplified with PCR using 200 nmol/l of S-D-Bact-0341-b-S-17/S-D-Bact-0785-a-A-21 primers (Klindworth et al., 2013) with Illumina overhang adapter sequences, in a final volume of 25 μl containing 12.5 ng of genomic DNA and 2X KAPA HiFi HotStart ReadyMix (Kapa Biosystems). PCR reactions were performed in a Thermal Cycle T gradient (Biometra GmbH) using the following thermal program: 3 min at 95 °C for the initial denaturation, followed by 25 cycles of denaturation at 95 °C for 30 s, annealing at 55 °C for 30 s, extension at 72 °C for 30 s, and a final extension step at 72 °C for 5 min. PCR products of about 460 bp were purified using a magnetic bead-based system (Agencourt AMPure XP; Beckman Coulter) and sequenced on Illumina MiSeq platform using the 2 × 250 bp protocol, according to the manufacturer's instructions (Illumina). The libraries were pooled at equimolar concentrations, denatured and diluted to 6 pmol/l before loading onto the MiSeq flow cell. Sequencing reads were deposited in the National Center for Biotechnology Information Sequence Read Archive (NCBI SRA; BioProject ID PRJNA591560).

### 2.5 Bioinformatics and statistics

Raw sequences were processed using a pipeline combining PANDAseq (Masella et al., 2012) and QIIME (Caporaso et al., 2010). After length (minimum/maximum, 350/550) and quality filtering (Phred score, 33), the UCLUST software (Edgar, 2010) was used to bin high-quality reads into operational taxonomic units (OTUs) through an open-reference strategy at a 0.97 similarity threshold. Taxonomy was assigned using the RDP classifier and the Greengenes database as a reference (release May 2013). Chimera filtering was performed discarding all singleton OTUs. Alpha rarefaction was evaluated by using the Observed OTUs metric, and the Shannon biodiversity index, which measures diversity based on evenness, while beta diversity was estimated according to the Jaccard similarity. Random Forests and all statistical analysis were computed using R software (version 3.1.3) and the packages randomForest (Breiman, 2001), vegan and made4. The significance of data separation on the PCoA was tested using a permutation test with pseudo*-F* ratios (function adonis of vegan package). Non-parametric and correlation tests were achieved with Wilcoxon rank-sum or Kruskal-Wallis test and the Kendall tau, respectively. Cortisol and testosterone concentrations, as well as T/C ratio was analyzed using the normality test of Shapiro-Wilk, in order to establish the distribution of each variable in the population. P-values < 0.05 were considered statistically significant.

## 3 Results

### 3.1 Relative abundance of major gut microbiome components in the enrolled cohort

In order to evaluate possible differences in GM communities among the behavioral study groups, we collected fecal samples from 42 dogs. In particular, 23 males and 19 females of different breed, aged between 1 and 13 years, were recruited from three animal shelters located in the metropolitan area of Bologna (Italy). Following the behavioral evaluation performed by a behaviorist veterinary and a dog handler, dogs were grouped based on their behavioral phenotype: 18 were classified as aggressive and 11 as phobic, while 13 exhibited a normal behavior. The 16S rRNA sequencing, performed using the Illumina MiSeq platform, yielded a total of 1,343,883 sequences (31,997 ± 5,454 sequences per sample), and 86% of the sequences have passed the length and quality filtering processes. High-quality sequences were subsequently clustered in 7,251 OTUs with a 97% identity threshold.

The most abundant phyla detected within the normal behavior samples were Firmicutes (relative abundance ±sd, 68.0 ± 4.6%), Bacteroidetes (13.7 ± 3.6%), and Actinobacteria (9.9 ± 1.6%), with Fusobacteria (4.8 ± 1.3%) and Proteobacteria (2.1 ± 0.8%) as minor components. The aggressive group showed similar proportions among the dominant phyla when compared to the normal behavior group, except for a reduced relative abundance of Bacteroidetes (P-value = 0.02; Wilcoxon test). No significant differences at phylum level were detected between the phobic and the normal behavior groups, as well as between the phobic and the aggressive groups.

**Figure 1:**
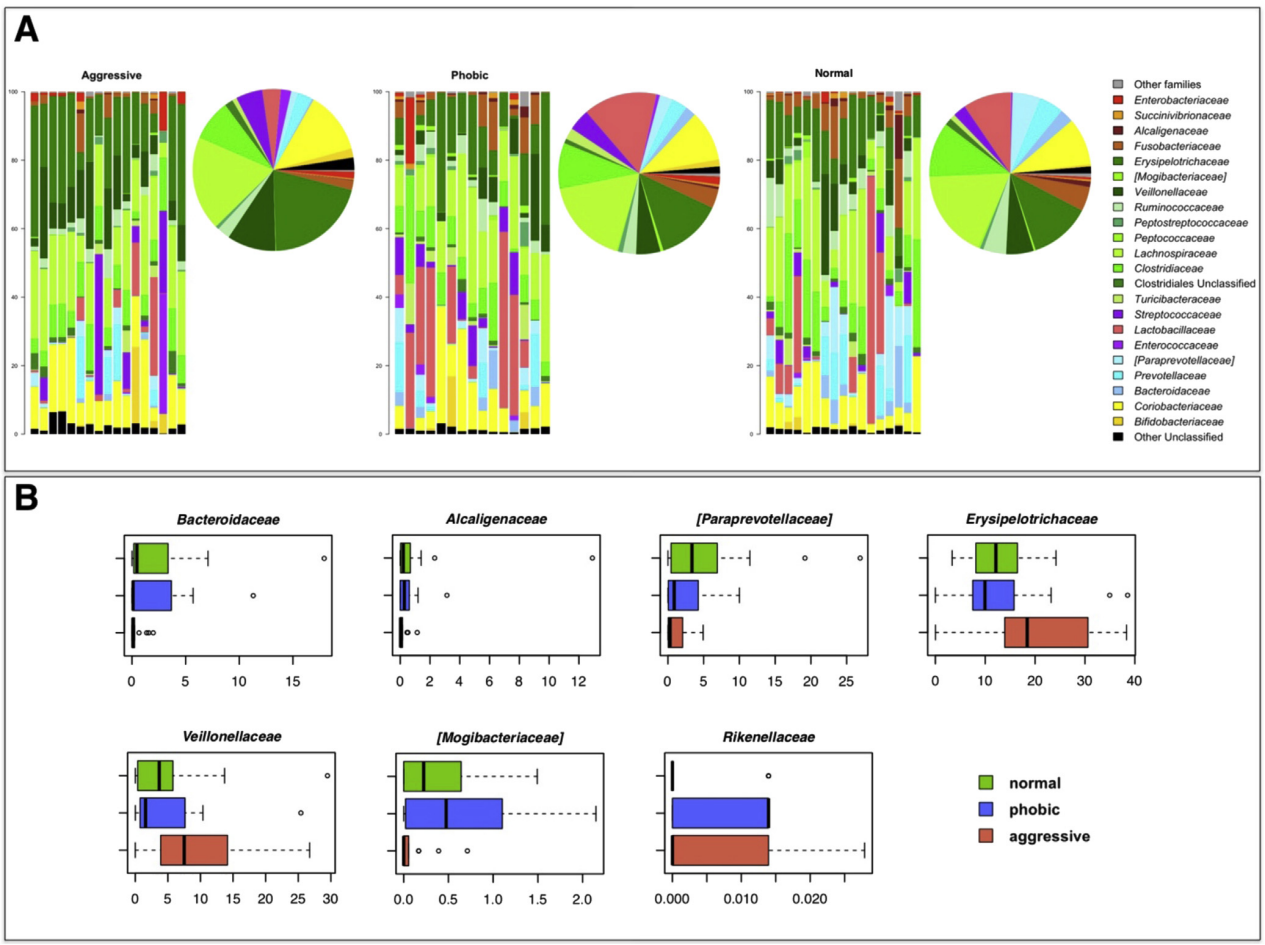
Canine gut microbiome profile of the behavior groups. (A) Relative abundances of familylevel taxa in each subject of the enrolled cohort (barplots) and respective average values of each study group (piecharts). Only bacterial families with relative abundance > 0.1% are shown. (B) Boxplots showing the distribution of the relative abundances of bacterial families significantly enriched or depleted within the gut microbiota of aggressive or phobic groups (P-value < 0.05, Wilcoxon).

At family level, Lachnospiraceae*,* Erysipelotrichaceae and Clostridiaceae constitute the major components of the normal behavior group (relative abundance >10%). A depletion in the relative abundance of Bacteriodaceae, Alcaligenaceae and [Paraprevotellaceae], as well as an increase in Erysipelotrichaceae (P-value < 0.05) was observed in the aggressive group compared to the normal behavior group. The phobic group was instead characterized by an increase in the relative abundance of the family Rikenellaceae (P-value = 0.04) when compared to the normal behavior group. Aggressive and phobic groups were found to be distinguishable due to different proportions in the relative abundance of the bacterial families [Mogibacteriaceae] and Veillonellaceae, respectively depleted and enriched in the aggressive group (P-value < 0.04) (Figure 1A, 1B).

At the genus taxonomic level, *Clostridium*, *Lactobacillus*, *Blautia* and *Collinsella* represent the major portion of the normal behavior group GM (relative abundance >5%). Several microbial genera were found to be significantly depleted in the aggressive group. The relative abundance of the genera *Oscillospira*, *Peptostreptococcus*, *Bacteroides*, *Sutterella*, and *Coprobacillus* were significantly lower in aggressive compared to normal behavior group, while *Catenibacterium*, *Megamonas* and [*Eubacterium*] showed an opposite trend (P-value < 0.04). At the genus level, no significant differences were detected between the phobic and the normal behavior group. The differences found between phobic and aggressive groups were due to an increased relative abundance of *Catenibacterium* and *Megamonas* in the latter group (P-value < 0.007), in addition to a slightly depletion of the genus *Epulopiscium* (P-value = 0.04) (Fig. S1).

Comparison of the overall gut microbiome compositional structure between aggressive, phobic and normal behavior dogs.

**Figure 2:**
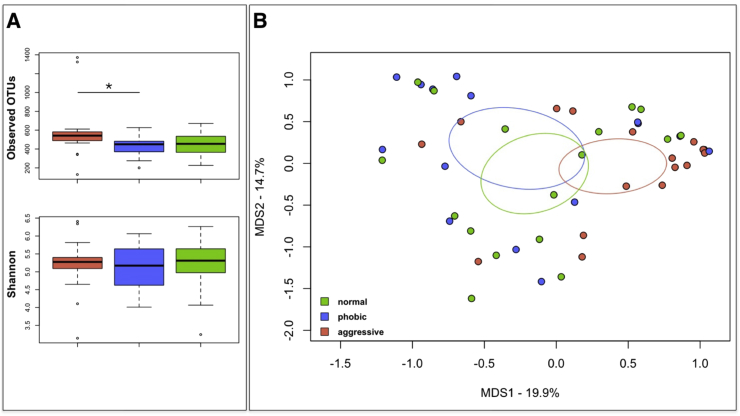
Biodiversity of the canine gut microbiome. (A) Boxplots showing the alpha diversity measures computed with phylogenetic and non-phylogenetic metrics (Shannon diversity index, observed OTUs). Behavior-related groups are identified with colored box and whiskers (orange, Aggressive; blue, Phobic; green, Normal). Significant difference was found between aggressive and phobic groups, according to the observed OTUs metric (P-value = 0.02; Kruskal-Wallis test). (B) Principal coordinate analysis (PCoA) plots showing the beta diversity of the intestinal bacterial communities of the study groups, based on Jaccard similarity index. A significant separation between aggressive and phobic behavior groups was found (P-value = 0.02, permutation test with pseudo-Fratios).

The intra-individual diversity of the canine GM was assessed by means of the metric Observed OTUs and the Shannon biodiversity index at the genus level. While according to the Shannon index the intra-individual GM diversity was comparable between aggressive, phobic and normal behavior groups (mean ± SD, 5.2 ± 0.8, 5.14 ± 0.7, and 5.2 ± 0.7, respectively), the Observed OTUs metric highlighted a statistical significant difference between aggressive and phobic groups (P-value = 0.02; Kruskal-Wallis test). In particular, the aggressive group was characterized by the higher number of different taxa observed (mean ± SD, 583.9 ± 310.6), the phobic group had the lower value (430.07 ± 112.28), and the normal behavior group exhibited intermediate values (454.8 ± 118.3) (Figure 2A).

According to the Jaccard similarity index, the Principal Coordinates Analysis (PCoA) of the inter-sample variation highlighted a significant separation between the structural composition of the GM among study groups (P-value = 0.02, permutation test with pseudo-*F* ratios) (Figure 2B). In order to identify bacterial drivers that contribute to groups clustering (permutation correlation test, P-value < 0.001), a superimposition of the genus relative abundance was performed on the PCoA plot. As showed in Fig. S2, major drivers of the normal behavior group segregation were *Faecalibacterium*, *Bacteroides*, *Phascolarctobacterium, Fusobacterium*, *Prevotella*, and [*Prevotella*]. The phobic group was characterized by an enrichment in *Lactobacillus*, while *Dorea*, *Blautia*, *Collinsella*, [*Ruminococcus*], *Slackia*, *Catenibacterium*, and *Megamonas* were more represented within the aggressive group.

**Figure 3:**
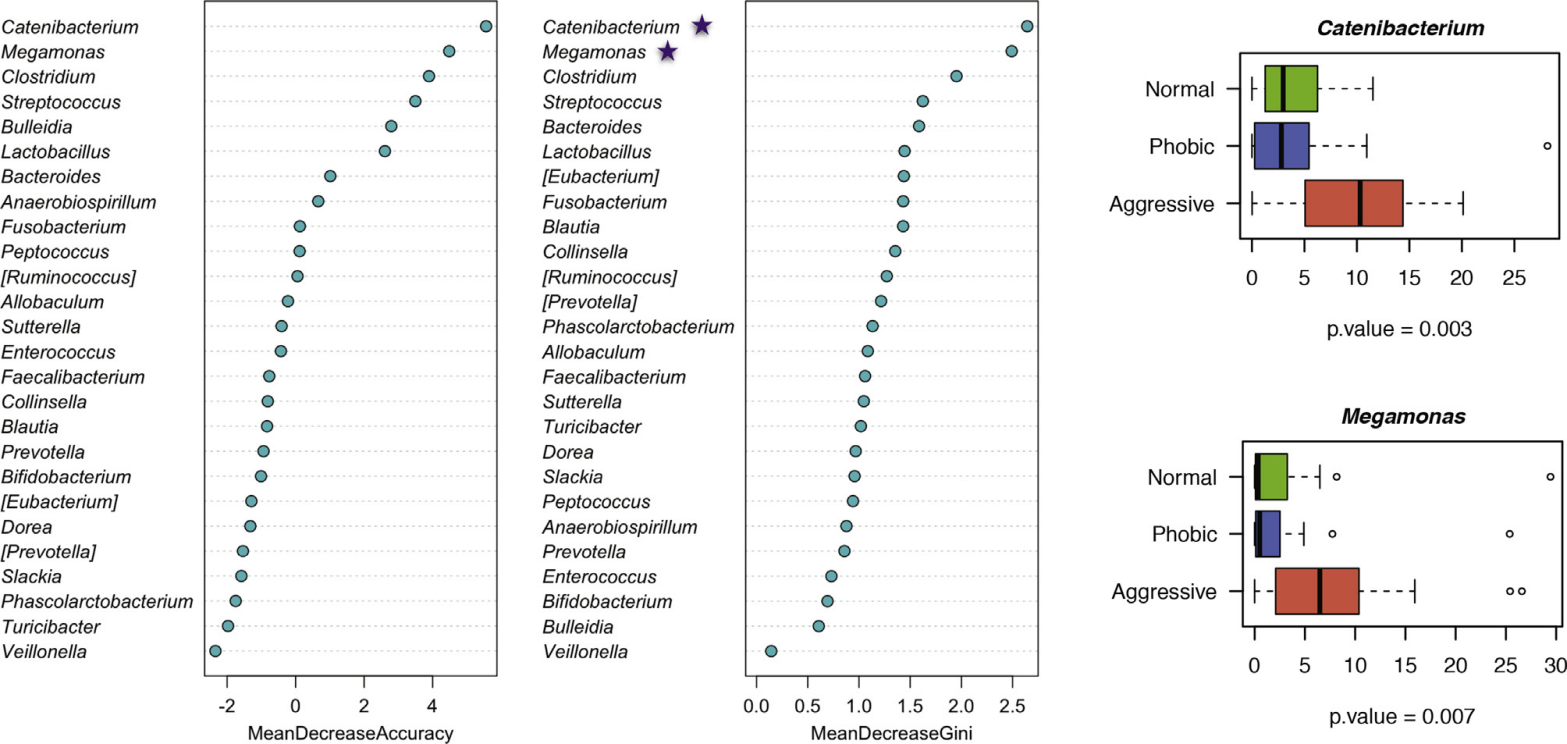
Behavior-related gut microbiome signature. Top 26 features from the obtained dataset as revealed by Random Forest. Stars denote the bacterial genera discriminant of aggressive group. Boxplots shows the comparison of the relative abundances of these bacterial genera between the study groups.

To dissect discriminant GM component for dogs showing aggressive behavior, we applied the machine learning method Random Forest (Breiman, 2001) to the genus level data set. Behavior-discriminatory bacterial genera were identified with distinctive changes in their relative abundances (Figure 3). Specifically, our analysis revealed two ‘aggressive-discriminatory’ bacterial genera: *Catenibacterium* and *Megamonas*.

### 3.2 Fecal cortisol and testosterone levels

**Figure 4:**
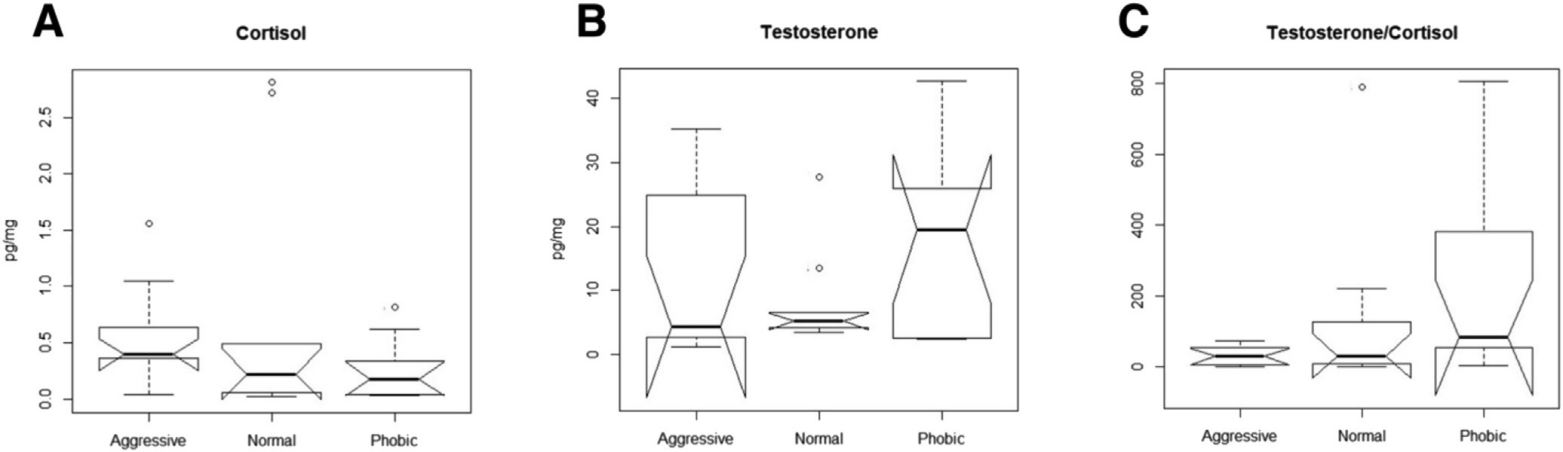
Fecal hormone levels of the behavior groups. . Boxplots showing levels of cortisol (A) and testosterone (B), and testosterone/cortisol ratio (C) detected in stool samples of the study groups. No significant difference was found among study groups (P-value > 0.05, Kruskal-Wallis test.

Cortisol and testosterone levels were measured in fecal samples through RIAs. The statistical analysis did not evidence any significant differences between the three groups of dogs for hormonal data. Our results showed that the three study groups had similar median value of fecal cortisol and testosterone levels (Figure 4A). However, it should be noted that the range of testosterone levels in aggressive and phobic populations was greater than that observed in the normal population, so we can assume that there is a greater variability among subjects with behavioral disorders (Figure 4B). As consequences of the previously described results, the median of testosterone/cortisol (T/C) ratio were similar in all the three groups, only slightly higher in phobic than in aggressive and normal dogs (Figure 4C).

## 4 Discussion

Within the present work, we profiled the GM structure and measured the fecal cortisol and testosterone levels of 42 dogs - 23 males and 19 females - of different breed, age, and weight, housed in individual cages of three animal shelters located in the metropolitan area of Bologna (Italy). Dogs were classified into three study groups based on their behavioral phenotype: aggressive, phobic or normal behavior. The phylogenetic profiles of the canine GM observed in our cohort were found to be in line with those already reported in literature for healthy dogs (Swanson et al., 2011; Sandri et al., 2017; Omatsu et al., 2018), but with a slightly higher abundance of Firmicutes and Actinobacteria, as well as a corresponding lower abundance of Bacteroidetes and Proteobacteria. According to our results, the aggressive group GM is characterized by a higher number of observed OTUs compared to both phobic and normal behavior groups. Interestingly, the GM structure of our cohort segregate according to the behavioral disorder of the host, showing a stronger separation of the aggressive group. The latter group seems to be defined by a higher abundance of typically subdominant taxa, such as *Dorea*, *Blautia*, *Collinsella*, [*Ruminococcus*], *Slackia*, *Catenibacterium*, and *Megamonas*. Conversely, the phobic group is characterized by an enrichment of *Lactobacillus*, a bacterial genus comprising well-known GABA producers, the main CNS inhibitory neurotransmitter able to regulate emotional behavior in mice via the vagus nerve (Lyte, 2011; Garcia-Mazcorro et al., 2012). The major drivers for the normal behavior group segregation are *Faecalibacterium*, *Bacteroides*, *Phascolarctobacterium*, *Fusobacterium*, *Prevotella* and [*Prevotella*], reflecting the predominance of bacterial genera commonly associated with the GM of healthy dogs (Farca et al., 2006; Vazquez-Baeza et al., 2016). Finally, according to the literature, no correlation was observed regarding the phobic behavior in relation to sex or age (Yalcin and Batmaz, 2007).

According to our results, fecal cortisol and testosterone levels of aggressive dogs did not significantly differ from those of phobic and normal dogs. Aggressive dogs are well known to possess higher blood concentrations of cortisol and lower serotonin levels than non-aggressive dogs (Rosado et al., 2010). However, fecal cortisol levels are not influenced by the activation of HPA axis during the sampling procedure, which is itself stressful for the animal (Archer, 1988). This can possibly explain the differences in the observed cortisol levels between studies carried out in blood or fecal samples, Testosterone is often correlate with aggressive behaviors in many species (Neilson et al., 1997), but this association is not completely demonstrated in dogs. Indeed, some studies have evidenced that the castration reduce only mildly aggressiveness and that neutered dogs can be more aggressive (Guy et al., 2001; Terburg et al., 2009). In contrast with results of Terburg et al. (2009), who suggested that a high value of T/C ratio may be a predictive factor of aggressive behavior, we found no significant differences between groups.

Studies about cortisol in phobic state are confusing, and it seems that cortisol does not increase, or increase only slightly in phobic subjects compared to normal ones (Fyer, 1998). Our results did not show any statistically significant differences between phobic and normal dogs for cortisol concentration. However, the cortisol level of phobic dogs is slightly lower than normal ones. Phobic state can cause a chronic and excessive stress (Schull-Selcer and Stagg, 1991), and scientific literature about phobic dogs suggests that they can tend to a depressive state. In human beings and in dogs, depression is characterized by lower cortisol and serotonin levels (Cocchi et al., 2013).

Our results suggest that dysbiotic GM configurations in a long-term stress levels scenario might influence the local gut environment through the release of potentially neuroactive microbial by-products, probably affecting the behavior of the host mainly as a side effect. In particular, dogs exhibiting aggressive behavioral disorders were characterized by a peculiar GM structure, a high biodiversity and an enrichment in generally subdominant bacterial genera. We then applied a machine learning method (Random Forest) to our genus level data set, identifying *Catenibacterium* and *Megamonas* as bacterial discriminants of aggressive behavior. Respectively belonging to the families Erysipelotrichaceae and Veillonellaceae, *Catenibacterium* and *Megamonas* have been recently correlated with primary bile acid metabolism and abdominal pain in humans (Sakon et al., 2008; Yusof et al., 2017; Aleman et al., 2018), suggesting a possible connection between a dysbiotic GM profile and behavioral disorders (McMillin and DeMorroe, 2016). We found no alteration within the GM of dogs with phobic behavioral disorder except for an increase in *Lactobacillus*, bacterial genera with well-known probiotic properties. Interestingly, chronic treatment with *L. rhamnosus* can influence anxiety- and depression-related behavior by modulating GABA receptor mRNA expression in specific brain regions (Bravo et al., 2011). Even if it is impossible to dissect the factors supporting the increase of *Lactobacillus* observed in phobic dogs, it is tempting to speculate that a higher abundance of this psychobiotics (Dinan et al., 2013; Sarkar et al., 2016; Nishida et al., 2019) could contribute to the establishment of peculiarities of the phobic behavioral phenotype. However, further studies are required to validate our findings. Indeed, the limitations due to the small number of enrolled animals imply a limited statistical power. Nonetheless, our study supports the intriguing opportunity that different behavioral phenotypes in dogs associate with peculiar GM layouts. Particularly, aggressive dogs possess dysbiotic GM configuration, possibly exacerbating the host aggressiveness and supporting the adoption probiotic interventions aimed at restoring a balanced host-symbiont interplay, improving the overall host health and eventually mitigating behavioral disorders.

## 5 Conclusion

Our results suggest that a long-term stress scenario influences the gut-microbiome composition. This preliminary research can thus be considered a starting point for future studies of clinical interest, which should deep the mechanisms underlying the relationship between behavioral disorders and the GM. Ultimately, this preliminary research provides new insights into veterinary behavioral medicine, which could help to develop a predictive diagnosis of canine behavioral disorders.

## Declarations

### Author contribution statement

Mondo E: Conceived and designed the experiments; Performed the experiments; Analyzed and interpreted the data; Wrote the paper.

Barone M: Performed the experiments; Analyzed and interpreted the data; Wrote the paper.

Soverini M, D'Amico F: Analyzed and interpreted the data.

Cocchi M, Accorsi PA: Conceived and designed the experiments; Contributed reagents, materials, analysis tools or data.

Petrulli C, Mattioli M: Contributed reagents, materials, analysis tools or data.

Marliani G: Analyzed and interpreted the data; Wrote the paper.

Candela M: Analyzed and interpreted the data; Contributed reagents, materials, analysis tools or data.

### Funding statement

This research did not receive any specific grant from funding agencies in the public, commercial, or not-for-profit sectors.

### Competing interest statement

The authors declare no conflict of interest.

### Additional information

Supplementary content related to this article has been published online at [URL].
