# Supplementary material for: Gut microbiome structure and adrenocortical activity in dogs with aggressive and phobic behavioral disorders
Source: Heliyon. 2020 Jan 29;6(1):e03311. doi: 10.1016/j.heliyon.2020.e03311 (PMC6994854; doi:10.1016/j.heliyon.2020.e03311)

S.1. Boxplots showing the distribution of the relative abundances of bacterial genera significantly enriched or depleted within the gut microbiota of aggressive or phobic groups (P-value < 0.05, Wilcoxon test).

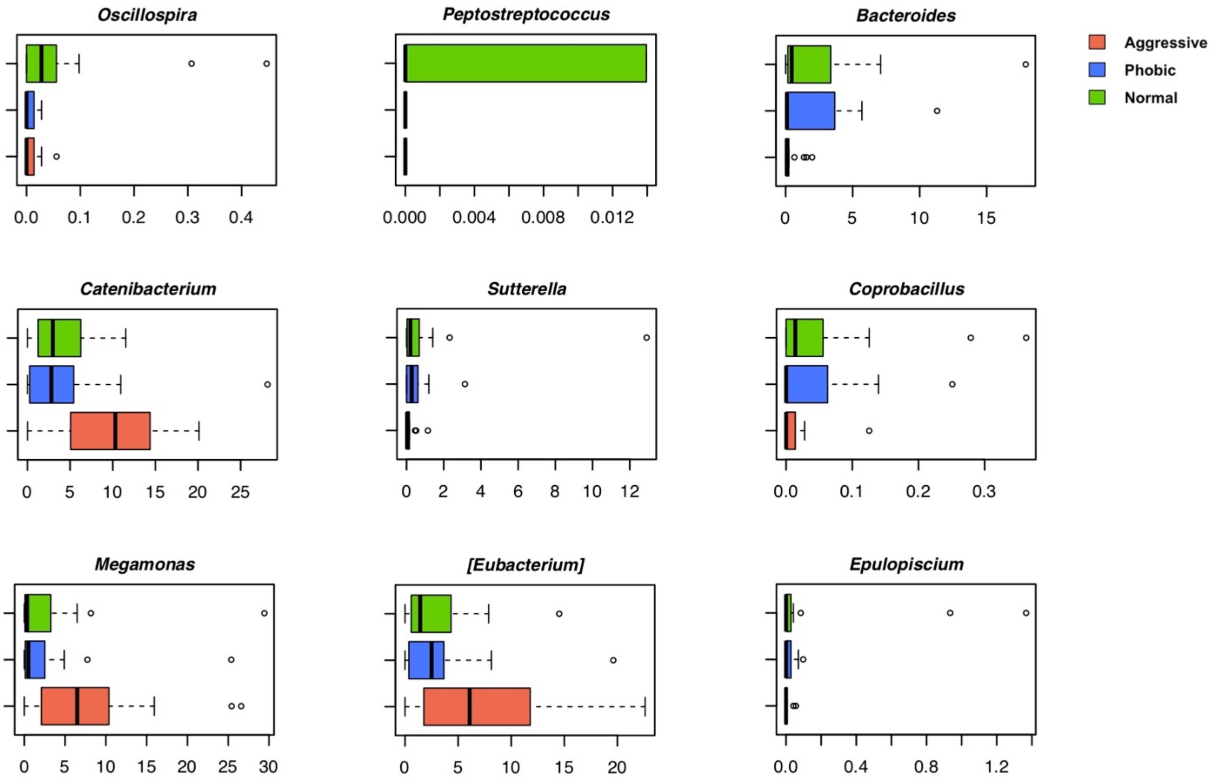

S.2. Superimposition of the genus-level relative abundance on the PCoA plot.

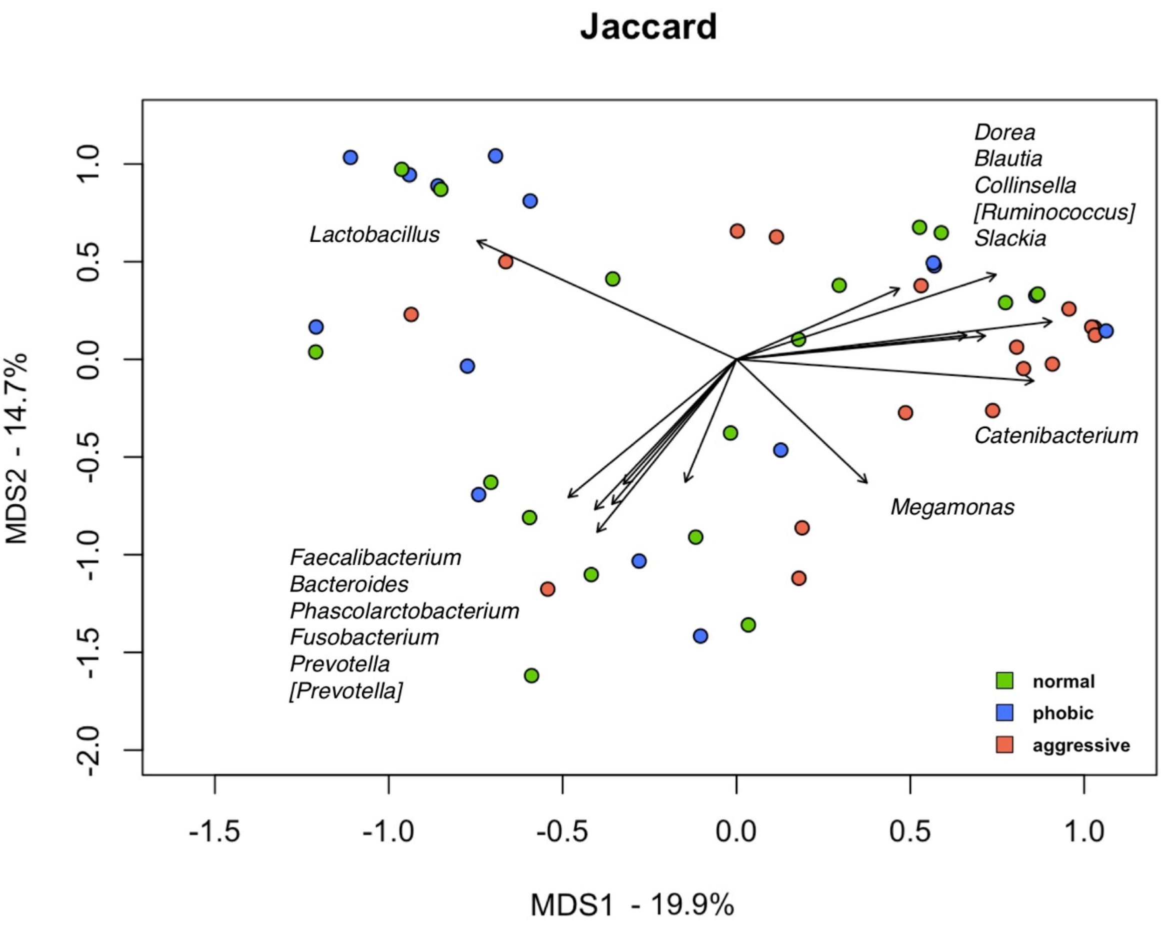

Supplement: Supplementary data [file mmc1.pdf]
